# Rare-earth- and aluminum-free, high strength dilute magnesium alloy for Biomedical Applications

**DOI:** 10.1038/s41598-020-72374-z

**Published:** 2020-09-28

**Authors:** Md Ershadul Alam, Soupitak Pal, Ray Decker, Nicholas C. Ferreri, Marko Knezevic, Irene. J. Beyerlein

**Affiliations:** 1 Departments of Mechanical Engineering, University of California, Santa Barbara, CA 93106 USA; 2 Materials Department, University of California, Santa Barbara, CA 93106 USA; 3 nanoMAG LLC, 13753 Otterson Ct, Livonia, MI 48150 USA; 4 Department of Mechanical Engineering, University of New Hampshire, Durham, NH 03824 USA

**Keywords:** Biomaterials, Structural materials

## Abstract

Lightweight, recyclable, and plentiful Mg alloys are receiving increased attention due to an exceptional combination of strength and ductility not possible from pure Mg. Yet, due to their alloying elements, such as rare-earths or aluminum, they are either not economical or biocompatible. Here we present a new rare-earth and aluminum-free magnesium-based alloy, with trace amounts of Zn, Ca, and Mn (≈ 2% by wt.). We show that the dilute alloy exhibits outstanding high strength and high ductility compared to other dilute Mg alloys. By direct comparison with annealed material of the same chemistry and using transmission electron microscopy (TEM), high-resolution TEM (HR-TEM) and atom probe tomography analyses, we show that the high strength can be attributed to a number of very fine, Zn/Ca-containing nanoscale precipitates, along with ultra-fine grains. These findings show that forming a hierarchy of nanometer precipitates from just miniscule amounts of solute can invoke simultaneous high strength and ductility, producing an affordable, biocompatible Mg alloy.

## Introduction

Being the lightest among the structural materials, and abundantly available in the earth’s crust and sea water, magnesium (Mg) and its alloys have regained interest from the scientific community after World War II, as an energy-efficient, environmental friendly, lightweight structural materials for many automobile, aviation, sport and electronic applications^1,2^. Also, as a class of biodegradable metals and having similar density and mechanical properties as natural bone, Mg-based alloys are being used as an implant material for the human body to support bone-tissue regeneration and a viable alternative to other biomaterials (eg., steel, titanium or cobalt) in use today^3–5^. Some limitations of current biomaterials are (a) the possible release of toxic metallic ions through wear or corrosion, (b) lower biocompatibility that causes tissue loss, and c) the need for a follow-up surgical procedure which adds cost and morbidity risks to the patient^3–5^. In addition, they have many other advantageous features, such as high specific mechanical properties, castability, machinability, weldability, and recyclability. While having many attractive attributes, pure Mg is, however, weak and suffers from low room temperature ductility or formability, limited high temperature strength, creep and corrosion resistance. These properties are currently holding Mg and its alloys back from widespread applications^1,2^.

Alloying with one or more elements (i.e. aluminum, zinc, silver, zirconium, calcium, rear-earth)^2,3,6–14^, compositing^1,2,8^, and varying thermomechanical processing parameters (i.e. solidification vs powder metallurgy, as-cast vs extrusion vs rolling, heat-treatments)^9,10,13,15,16^ are well known, successful methods for improving some properties of Mg. For example, Al additions help to improve strength, ductility and corrosion resistance^7,8^. However, due to the softening of the γ-Mg_17_Al_12_ precipitates at elevated temperatures, the Al-added Mg alloys exhibit poor-heat-resistance at/above 120ºC^17^. Furthermore, Al can be neurotoxic. Higher concentrations of Al^3+^ in the brain have been related to Alzheimer’s disease or found to hinder tissue growth^3,11,18^. Rare-earth (RE) elements are also often added to the Mg-based alloys for better formability and strength^13,16^. However, besides cost, some RE alloying elements have been shown harmful to the human body^4,19^.

Recently, Mg–Zn–Ca-based alloys, as well as those further micro-alloyed with Zr, Mn and other non-toxic elements, have received great interest as low-cost structural biocompatible materials^3,4,10,11,20,21^. These Mg–Zn–Ca alloys are biodegradable and moderately resistant to corrosion. With grain-boundary strengthening^22,23^, solid solution hardening^21^and precipitation hardening^9,10,12^, this alloy class can be made with relatively high strengths. The Ca addition helps to form Mg_2_Ca intermetallics that improve creep strength^10,24^. This solute also has been found to weaken the basal texture, characteristic of rolled Mg-based alloy, and thus, improve uniformity in properties and ductility^23^. However, Ding et al.^25^ reported that more than 1wt.% Ca can deteriorate the corrosion resistance. The Zn addition helps to strengthen this type of alloy both by solid solution and precipitation hardening^20,21,26^. However, while Zn has a high solubility limit (6.2 wt.%) in Mg, a recent study reported that excessive Zn additions can promote corrosion rates, due to the formation of Zn-rich intermetallic particles that act as cathodic sites^11,27^. Mn additions have the advantage of improving corrosion resistance^28,29^. In small amounts, further additions of Mn have been shown to improve the strength of these Mg–Zn–Ca based alloys by grain boundary strengthening effect^11,14^. Other studies have reported that Mn additions can lead to increases in ductility and reductions in grain size^30,31^. Another study has shown that too much Mn can, however, be toxic^32^. A similar outcome has been reported with further additions of RE elements, yet RE has issues as noted above^13,19^. Furthermore, in many of these cases, the combined fractions of alloying elements are too high to be used as implant materials^3,9–11,13–15,26,33^. The human body can tolerate only a small amount of non-toxic elements (such as, Zn, Mn, and Ca)^3,34^.

Dilute hexagonal close packed alloys do not exhibit uniform strength in all directions. Most of the Mg–Zn-based alloys, for instance, report the mechanical properties only for their strongest (generally rolling) direction, and often missing is the compressive response or responses in other sample directions. When the material is to be used in automobile or aviation applications, they will need to be deformed to their expected shape by a complex deformation path, and when they are designed as implant materials, then their compressive strength must also be assessed.

In this work, we report on an outstanding combination of mechanical properties obtained in an Mg alloy, BioMg250^35,36^, with only trace amounts of Ca, Mn, and Zn (in total approximately 2 wt.%). Including Zn and Ca together is important since they have a relative ratio that helps them to co-segregate to the grain boundaries and hinder grain boundary migration, resulting in a fine, nearly equiaxed grain structure^37^. At the same time, microalloying with a Zn/Ca ratio helps to minimize coarse anodic and cathodic intermetallic particles to tune the bio-absorption time in vivo^35,36^. We selected to use additional trace amounts of Mn for corrosion resistance^28,29^ and Ca for creep resistance^10^. To examine its strength, both the tensile and compression tests were performed and in three different directions of the sample. To identify the source of its strength at the nanoscale, we employed transmission electron microscopy (TEM), high-resolution TEM (HR-TEM) and atom probe tomography (APT), and post-annealing steps. The BioMg250 alloy is shown to achieve an optimal tensile yield and ultimate strength of 267 MPa and 307 MPa, respectively, and ductility of 21% and compression strength and strain to failure of 441 MPa and 8.5%, respectively. These properties are found to be outstanding when compared to numerous classes of other Mg alloys, particularly those that are also biocompatible material candidates. By repeating these tests on annealed material and a range of microscopy, we show that the outstanding combination of properties can be attributed to its hierarchical microstructure, including a bimodal grain structure including both submicron grains and large 10-micron grains, both grain boundary and grain interior precipitates, and a suite of Zn-containing precipitates, some just a few nm in dimension and others 10 nm in dimension. By virtue of its superior properties and its low-cost, RE-free, Al-free, dilute alloy composition, this dilute alloy is an ideal candidate for biomedical applications, as bioresorbable implants to fix bone trauma.

## Results

Two forms of the dilute, Mg-Zn-Ca-Mn alloy sheet were made. One form was peak-aged (PA) to encourage the formation of nanoscale coherent precipitates from its trace alloying elements, called Guinier–Preston (GP) zones^35,36^. The second form annealed (AN) begins with the same peak-aging step but is followed by an annealing step of 400ºC for 1 h to dissolve the GP zones. Composition and processing details can be found in the methods section. With 2 wt.% or less alloying elements, this alloy can undoubtedly be considered as dilute.

**Figure 1:**
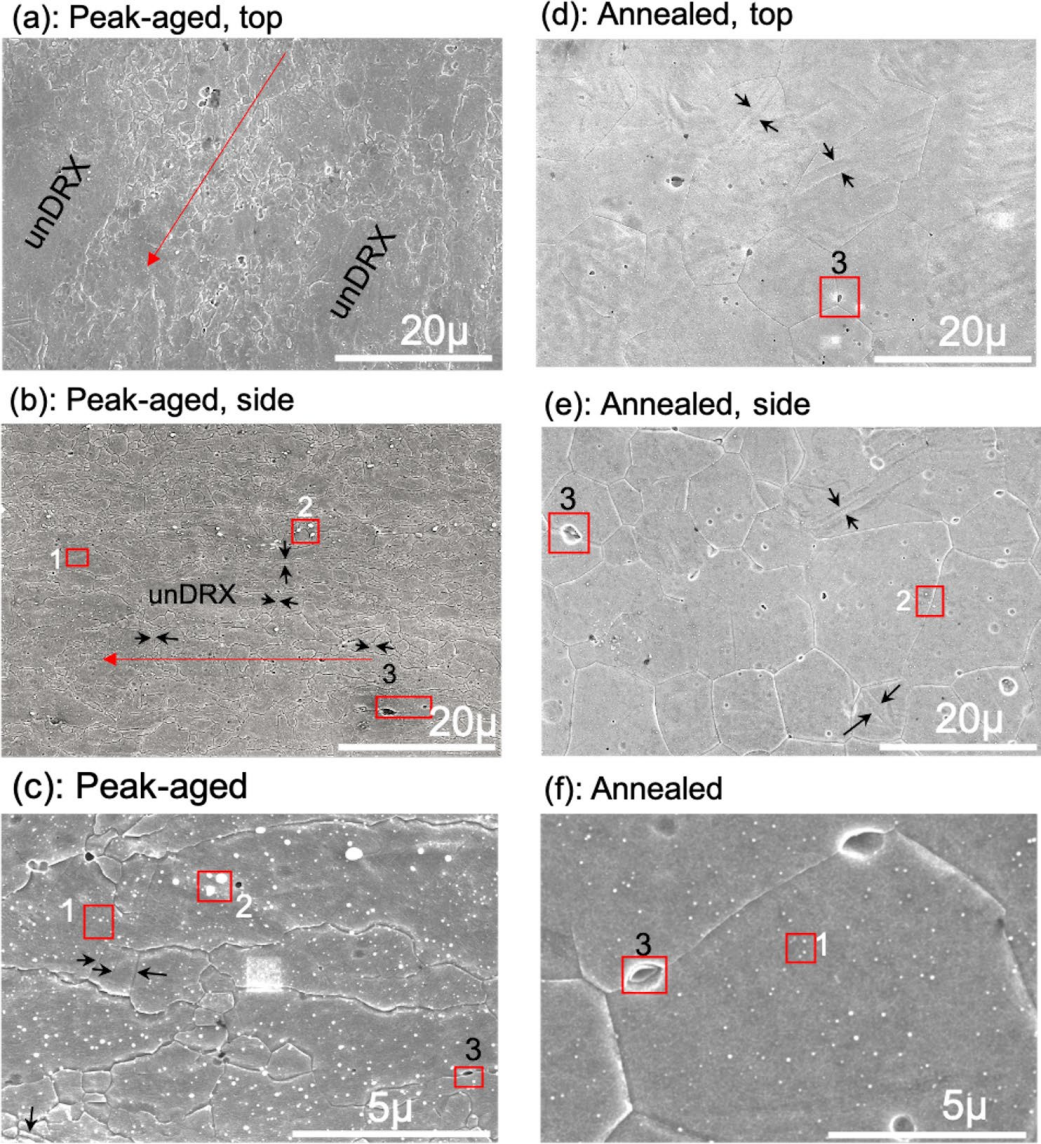
SEM images showing the grain morphology and precipitates for the (**a**–**c**) peak-aged material: (**a**) top view; (**b**) side view; and (**c**) higher magnification image to further show the submicron grains containing precipitates and twins. For the annealed material: (**d**) top view, (**e**) side view, and (**f**) higher magnification image again to show the precipitates. The red arrow indicates the rolling direction and the black arrows the twins. Labels 1, 2 and 3 in these images point respectively to the alpha-Mn precipitates, the Ca_2_Mg_6_Zn_3_ particles, and the Mg_2_Ca particles, which have been identified by the EDS scans in Figs. 5 and 6.

The strengths of these metals are affected by their grain size. Here, the morphology and size of approximately 500 grains in these two alloy forms is first assessed using SEM. This method provides for the widest field of view among the techniques used here. To obtain an approximate 3D perspective from the 2D sections, the grain morphology in both the top and side views of the plate were analyzed (see supplemental figure, SFig-1) and the results were nearly identical. The grain microstructure of the PA material is inhomogeneous with a bimodal grain structure. Approximately 95% of the grains are dynamically recrystallized (DRX) grains and below 5 µm in size. Among this group, nearly half are ultra-fine grains with an average grain size of 0.7 ± 0.2 µm (see Table 1 and Fig. 1a–c). The remaining 5% of the grains are un-recrystallized (un-DRX), relatively large grains, with an average size ≈ 20 µm and ranging up to ≈ 60 µm. The mean major-to-minor axis ratio (GAR), when considering all grains, increases with increasing grain size, indicating that the smaller DRX grains are nearly equiaxed and the larger un-DRX grains are elongated in the rolling direction.

**Table 1:** SEM grain morphology for peak-aged and annealed BioMg250.

Conditions	Range (µm)	Major length, l (µm)	Aspect ratio, GAR (l/s)	No. freq. of grains, (%)	Area fraction of grains, (%)
**Peak-aged**					
Top	0–1	0.7 ± 0.2	1.6 ± 0.4	47.4	4.2
	1–5	1.8 ± 0.8	2.1 ± 1.2	47.6	28.3
	5 +	20.7 ± 14.3	3.2 ± 1.7	5.0	67.5
Side	0–1	0.6 ± 0.2	1.7 ± 0.8	49.3	4.3
	1–5	2.0 ± 1.0	2.4 ± 1.1	43.9	30.2
	5 +	8.7 ± 3.4	4.9 ± 2.9	6.8	65.4
**Annealed**					
Top	0–1	–	–	–	0
	1–5	–	–	–	0
	5 +	13.3 ± 4.7	1.4 ± 0.4	100	100
Side	0–1	–	–	–	0
	1–5	3.7 ± 0.8	2.1 ± 2.0	8.5	2.6
	5 +	10.5 ± 3.3	1.3 ± 0.3	91.5	97.4

The annealed material is, in contrast, homogeneous, with a uniform grain size and shape distributions and the GAR is ≈ 1.3. Analysis of both the top and side views finds that its microstructure consists of fully recrystallized grains with an average size of ≈ 13 µm from the top view and ≈10 µm from the side-plate view (see Table 1 and Fig. 1d–f). The SEM scan reveals a few twin lamellae. Considering only the finer grains in the PA sample, the annealing step resulted in an order of magnitude larger grain size.

**Figure 2:**
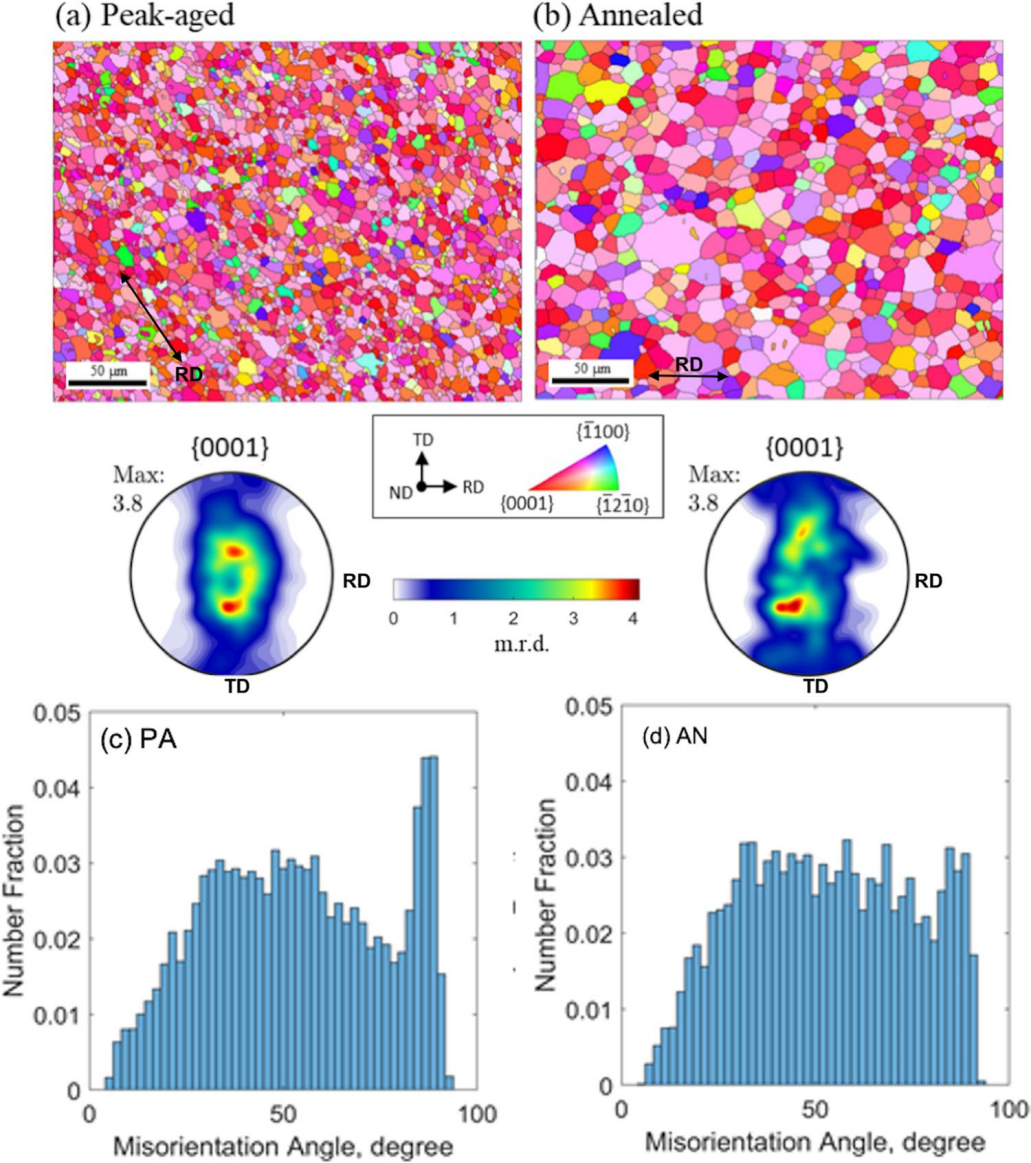
Inverse pole figure maps of the mean grain orientations and pole figures constructed from the EBSD data of the (**a**) peak-aged (PA) material and (**b**) annealed (AN) material. These confirm the dominance of the (0001) basal plane for both materials. There is a slight spread in the basal poles along the transverse direction as well as much larger grain sizes for the (**b**) AN material. The misorientation angle distributions for the (**c**) PA and (**d**) AN material.

The low symmetry of the hexagonal close packed crystal structure of Mg makes the strength sensitive to its texture, the distribution of lattice orientation among its grains, and possibly misorientation between neighboring grains and twins. Figure 2a, b shows the textures in the form of basal pole figures obtained using electron backscattered diffraction (EBSD). The data are taken from 300 × 300 μm EBSD scans in the ND cross-section, or top view. At this measurement scale, the two materials have nearly the same texture. Their main features are a dominant (0001) type basal texture, preferentially oriented through the thickness of the sheet, and a spread along the transverse direction (TD). Both textures are notably weak compared to most conventionally rolled Mg alloys. The maximum intensity of the basal pole figures here is 3.8 m.r.d., which is relatively low. Weak textures are desired as it implies that the texture contribution to plastic anisotropy will be reduced^38,39^. One of the desirable aspects of RE-Mg alloys are their characteristically weak textures^16,40^, similar in intensity to the dilute, non-RE Mg material shown here. Many available Mg alloys, such as AZ31 series^40–42^ or even some dilute Mg–Ca/Mg–Zn alloys^43^, typically have maximum intensities of 10 m.r.d. or greater.

Figure 2c, d shows the distributions of grain misorientation angles for both materials. As in the bulk textures, there are no significant differences in their misorientation distributions. The distribution is broad, ranging from 5 to 90 and the average misorientation angle is 53º in both cases. There is some intensity at a misorientation angle of 86.3º in both materials, being more pronounced in the PA material. This peak misorientation corresponds to the theoretical twin/matrix misorientation angle of 86.3º of the {1012} twin in Mg, and can be associated with the few twin lamellae identified from the SEM analysis of the both PA and AN samples (see black arrows in Fig. 1). This {1012} twin type is the most common among Mg alloys and was inherited from the rolling step used in material preparation^38^. To summarize, the post-annealing step has effectively homogenized the grain structure and increased the average grain size by an order of magnitude, while mostly preserving the initial texture and grain boundary misorientation distribution.

**Figure 3:**
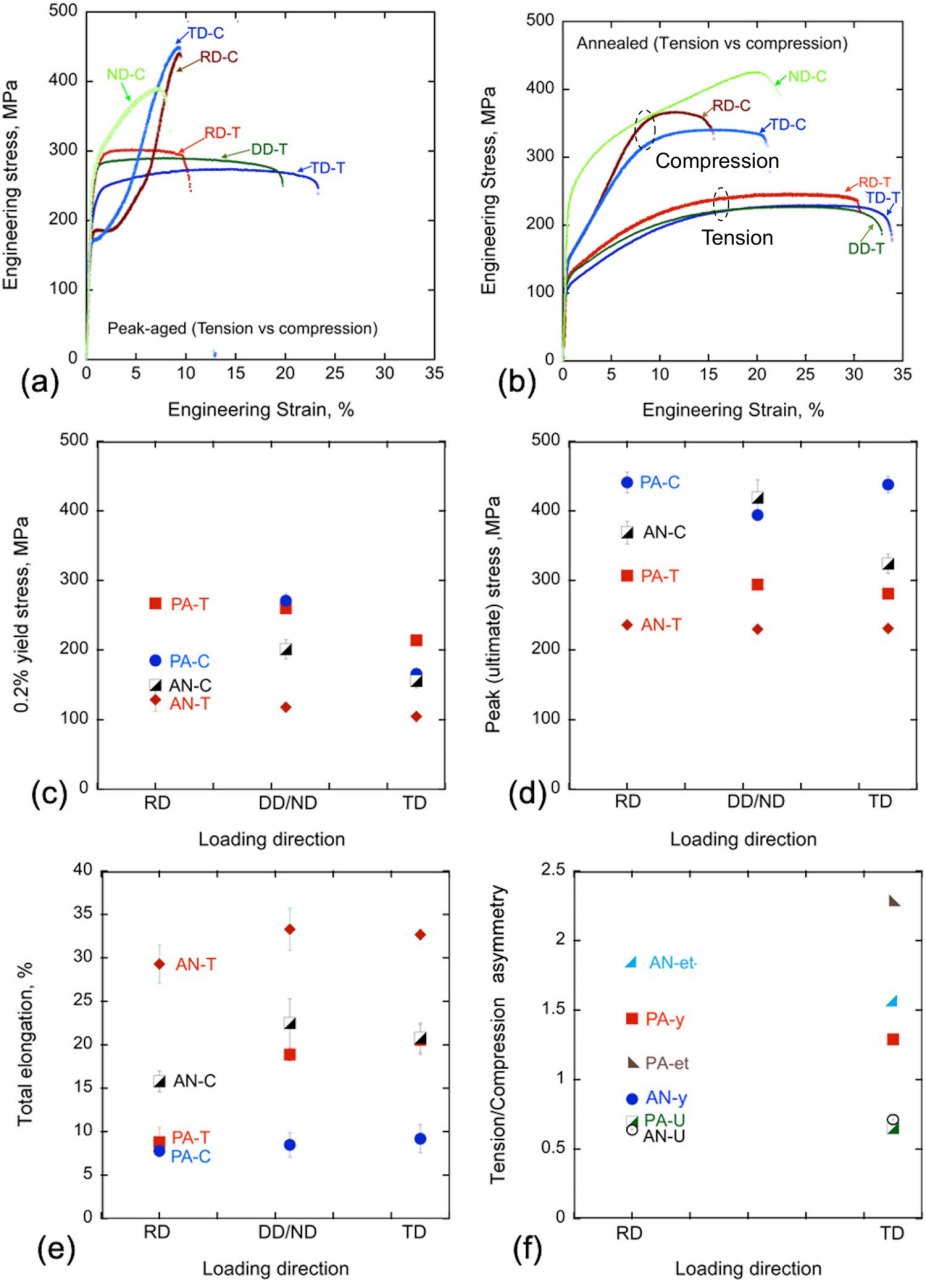
Room temperature engineering stress–strain (s–e) curves in tension and compression for the (**a**) peak-aged and (**b**) annealed samples. (**c**)–(**e**) show their 0.2% yield stress, peak or ultimate strength, and elongation to failure, respectively. (**f**) the tension/compression asymmetry in the peak-aged and annealed samples.

Figure 3 shows the tensile stress–strain responses of the PA material in three in-plane directions (see SFig-1), from which the yield stress, ultimate stress, and elongation to failure (ductility) can be extracted. The yield (s_y_) and ultimate stress (s_u_) in the RD are highest being 267 MPa and 307 MPa, and slightly lower in DD, 260 MPa and 294 MPa, and lowest in the TD, with 214 MPa and 281 MPa (see Table 2 and Fig. 3a,c). The loading direction dependence in these strengths is a consequence of the preferred basal texture (see basal pole figure in Fig. 2), wherein the c-axis distributions are not axisymmetric, but spread along the TD. The resulting tensile plastic anisotropy is often seen in pure Mg and other Mg alloys, wherein RD-tension requires higher contributions of the harder prismatic slip and less of the easier basal slip compared to those activated during TD or DD-tension^44–46^. Because some of the precipitates found in this alloy lie preferentially on the basal plane, they tend to harden the prismatic slip mode, thereby further enhancing the tensile anisotropy. The loading direction dependence in these strengths is a consequence of the preferred orientation of the c-axis through the thickness of the sheet (see basal pole figure in Fig. 2). The c-axis distributions are not axisymmetric, with a spread along the TD.

**Table 2:** Room temperature tensile properties of peak-aged and annealed BioMg250 compared to many other Mg alloys in the literature^3,8–16,26,27,42,47–58,60–62^.

Alloys	Processing cond^n^ + loading dir	0.2% yield strength, s_y_ (MPa)	Peak/ultimate strength, s_u_ (MPa)	Uniform elongation, e_u_, %	Total elongation, e_t_, %	Reduction of area, RA, %; [Ref]
BioMg250-PA	Rolled_RD	267 ± 3	307 ± 1	4.8 ± 0.9	8.8 ± 1.7	13 ± 0
	Rolled_DD	260 ± 4	294 ± 2	6.2 ± 1.3	18.9 ± 0.7	25 ± 2
	Rolled_TD	214 ± 6	281 ± 6	13.4 ± 1.5	20.6 ± 1.7	27 ± 2
BioMg250-AN	Rolled_RD	129 ± 17	236 ± 5	19.7 ± 1.8	29.3 ± 2.2	32 ± 5
	Rolled_DD	118 ± 1	230 ± 1	22.3 ± 1.4	33.3 ± 2.4	34 ± 5
	Rolled_TD	105 ± 1	231 ± 1	23.7 ± 3.1	32.7 ± 0.6	30 ± 4
Al-added alloys						
AZ31	Rolled_RD	160	220	–	10	^42^
AZ41	Ext_ED	218 ± 5	287 ± 6		8.2 ± 0.3	^8^
AZ51	Ext_ED	222 ± 4	302 ± 4		8.7 ± 0.4	
AZ61	Ext_ED	230	310		16	^47^
AZ91	Ext_ED	272 ± 3	353 ± 0	–	3.7 ± 0.5	^48^
	Cast + T6	120	200	–	2	^49,50^
Mg–8.0Zn–1Al–0.5Cu–0.6Mn	Cast + T6	228	328		16	^15^
Mg–6Zn–1Mn–3Al	Rolled + T6	319	360		6.3	^12,51^
Mg–3.5Al–3.3Ca–0.4Mn	Ext	410	420		6	^12^
RE-added alloys						
Mg–2.5Zn–2.5Y–1Mn–(0–0.67) Ca	As-cast	–	191–231		4.8–8.6	^14^
Mg–8.2Gd–3.8Y–1Zn–0.4Zr	Rolled + T5	426	517		4.5	^13^
Mg–1Ca–1Zn–1Nd–0.6Zr	Cast + T6	153	227		7	^50^
WE43	T5, RD	210	347		15	^16^
	T5, TD	230	342		12	
Al- and RE-free Mg–Zn–Ca–Mn alloys with > 2 wt.% elements						
Mg-4Zn	Cast and HT	129	253	–	11	^10^
Mg-4Zn–0.1Ca		135	263	–	11	
Mg–4Zn–0.5Ca	Cast + HT	60	211		17	^52^
	Ext (320C)	170	273		34	
Mg–4.7Zn–0.5Ca	Ext	291	329		16	^12^
Mg–6Zn	Ext, ED	125 ± 5	276 ± 1	–	29.7 ± 2.7	^53^
Mg–6Zn–0.4Ca		169 ± 4	276 ± 3	–	21.4 ± 1.1	
Mg–6Zn–0.8Ca		230 ± 8	304 ± 1	–	15.3 ± 1.6	
Mg–6.4Zn–0.2Ca–0.2Mn	Ex 300C	202	287		24	^9^
	Ex 270C	253	309		22	
	Ex 270 + T5	290	304		22	
Mg–2Zn–1Mn–(0.3–1.0) Ca	As-cast	60–80	140–180		2–8	^3^
Mg–4Ca	As-cast	34	77		2.1	^11^
Mg–2Ca–0.5Mn–(2–7)Zn		45–83	140–190		4.1–8.7	
Mg–0.2Ca–2.5Zn	As-cast As-cast	78	135		3.1	^26^
Mg–0.2Ca–2Zn		68	170		6.5	
ZK60 (Mg–6Zn–0.5Zr)	As-ext, ED	237	312		15.5	^54^
	T5, ED	273	329		16.5	
Mg–1Ca–1Zn–0.6Zr	Cast + T6	145	241		9	^50^
	Ext (350C)	306	314	–	10.3	^12,55^
	Ext (400C)	262	291	–	11.7	
Al-and RE-free dilute Mg (≤ 2 wt.%)						
Mg–0.1Ca	Ext (230C)	290 ± 5	300 ± 4		13	^56^
Mg–1Ca	Ext (230C)	377 ± 5	392 ± 6		2	
Mg–2Ca	As-cast	47	115		3	^11^
Mg–0.1Ca	Rolled + T5_RD	99	168		16	^57^
Mg–0.4Ca		102	175		7.4	
Mg–0.4Zn		93	186		15.5	
Mg–0.3Zn–0.1Ca		93	183		24	
Mg–0.5Zn–0.1Ca	Rolled + HT_RD	132	207		23	^58^
Mg–1.5Zn–0.1Ca		120	220		23	
Mg–0.2Zn–0.5Ca	Ext (350C)	64 ± 5	184 ± 11	15 ± 5	16 ± 5	^60^
	Ext + HPT (2 rot) + 1 h/142C	237 ± 10	308 ± 20	1.0 ± 0.6	1.1 ± 0.7	
Mg–0.6Zn–0.5Ca	Ext (350C)	117 ± 5	237 ± 3	14 ± 2	15 ± 3	
	Ext + HPT (2 rot) + 1 h/142C	216 ± 16	238 ± 33	0.4 ± 0.4	0.4 ± 0.4	
Mg–1Zn–0.3Ca	Ext (325C)	247	268		20	^27^
Mg–0.3Al–0.2Ca–0.5Mn	Ext + T5	207	250		12.5	^60^
Mg	ED	124 ± 11	201 ± 13	–	6.1 ± 1.1	^8^
Al-6063	T6	215	240	–	12	^61^
Al-AA6xxx	T4	120–160	220–290	–	24–30	^62^

Considering results from all test directions, these strengths are remarkably high. The intermediate DD strength, along with its large ductility (e_t_) of 19%, are higher than those of many other more heavily concentrated Mg-Zn-Ca alloys^3,10,11^. The DD strength is also greater than that of AZ31, a commercially popular structural Mg alloy made with Al.

To determine tension–compression (T-C) asymmetry, the same sheet material was also tested in compression in the TD and RD directions. Since compression samples can be smaller than tension samples, compression tests were also conducted in the ND direction. Figure 3 shows the resulting stress–strain curves. The yield stress and peak/ultimate strengths in compression ranged from 166 to 438 MPa for the TD to 271 to 394 MPa for the ND, respectively (see Table 3 and Fig. 3a,c). Compared to compression strengths of other Mg alloys, even those with higher solute concentrations, the compression strengths of the present alloy are higher. The tension–compression asymmetry is defined as the ratio of tensile to its respective compressive properties (i.e. yield, peak or deformation to failure). In the TD and RD, the T-C asymmetry in the yield are 1.29 and 1.44 and peak strengths are 0.64 and 0.7, respectively (see Tables 2 and 3, Fig. 3f). The T–C asymmetry in the deformation to failure is 2.24 for TD and 1.13 for RD. The compression yield and strain hardening for ND are notably higher than those in the RD or TD for both conditions. This plastic anisotropy in compression is commonly seen in rolled pure Mg and dilute Mg alloys, wherein the preferred orientation of the c-axis through the thickness of the sheet promotes the harder pyramidal slip in ND compression and the easier {1012} tensile twinning in RD and TD compression^44–46^.

**Table 3:** Room temperature compression properties of BioMg250.

Conditions [Ref]	Loading directions	0.2% compressive yield stress, cs_y,_ (MPa)	Compressive ultimate strength, cs_u_ (MPa)	Compressive uniform elongation, ce_u,_ (%)	Compressive total elongation, ce_t,_ (%)
BioMg250-PA	RD	185 ± 4	441 ± 15	7.6 ± 0.7	7.8 ± 0.6
	ND	271 ± 10	394 ± 2	7.1 ± 1.1	8.5 ± 1.4
	TD	166 ± 6	438 ± 12	9.1 ± 1.5	9.2 ± 1.6
BioMg250-AN	RD	150 ± 5	369 ± 16	10.8 ± 1.6	15.8 ± 1.2
	ND	201 ± 14	419 ± 26	20.4 ± 2.8	22.5 ± 2.8
	TD	156 ± 10	324 ± 14	13.8 ± 2.9	20.8 ± 1.7
Mg–6.4Zn–0.2Ca–0.2Mn^9^	ED-300C	184	470	17	24
	Ext at 270C	235	491	13	
	270C + T5	269	484	13	
AZ31^42^	RD	115	458	–	17
WE43^16^	T5-RD	250	496	–	14
	T5-TD	270	499		12

**Figure 4:**
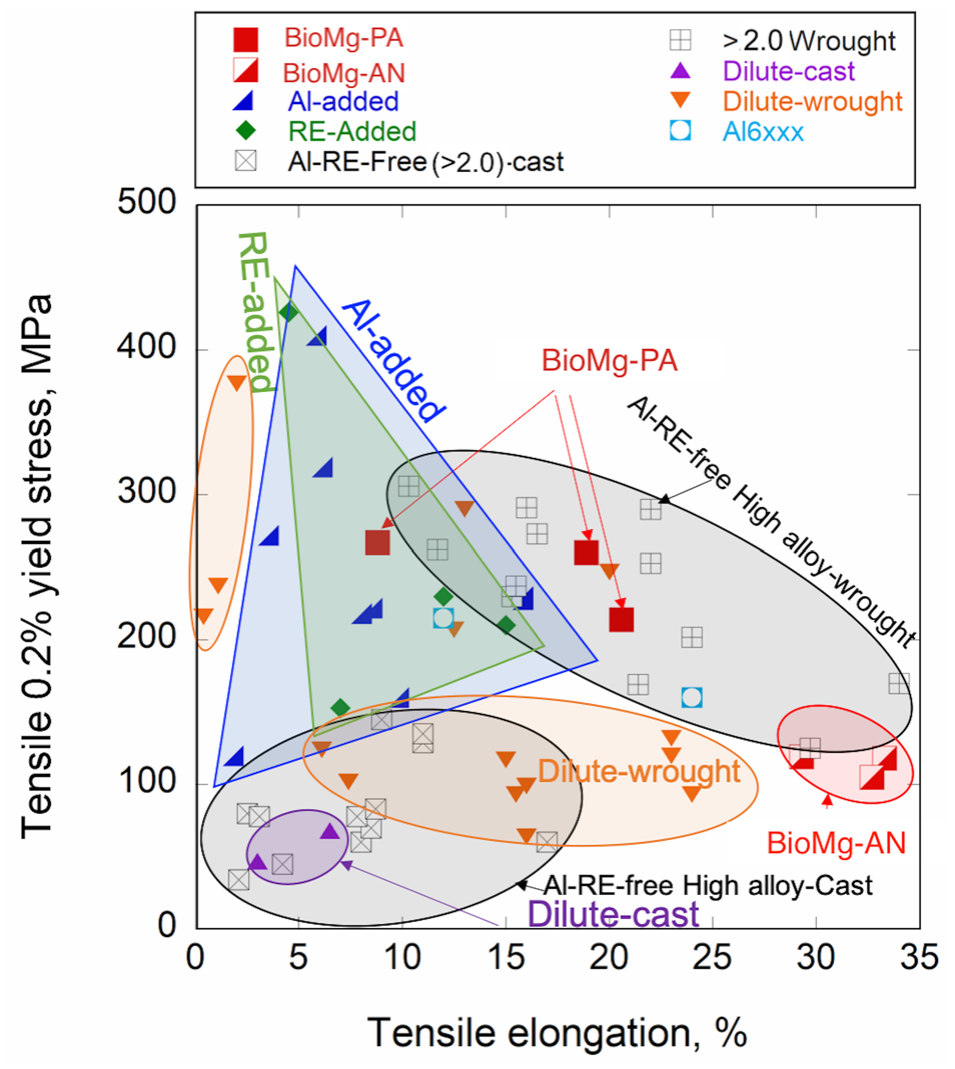
Plot compares the 0.2% tensile yield versus elongation for the present dilute Mg alloy with many other types of Mg-based alloys in the literature^3,8–16,26,27,42,47–62^.

To demonstrate the superior strengths of this alloy, Fig. 4 compares its strength-elongation strain to several classes of Mg alloys, including those with higher concentrations of solutes than this alloy (labeled “high alloy”), or containing Al, or RE alloying elements^3,8–16,26,27,32,43–60^. For BioMg250, we include the yield and ultimate tensile strength and ductility in all three directions. As shown, this light concentration, biocompatible alloy achieves an excellent balance of strength and ductility compared with these other classes of Mg alloys. Specifically, regarding strength, BioMg250 is stronger compared to similarly dilute or lightly concentrated Mg alloys. The material is also stronger than the 6xxx series Al (Mg–Si–Cu) alloys which are widely regarded as the structural materials for automobile applications^61,62^. The BioMg250 alloy is, however, weaker (but more ductile) than the AZ91^48^ or Mg-6Zn-1Mn-3Al Mg alloys that contains Al^12,51^, or higher concentrations of alloying elements, such as Mg–4.7Zn–0.5Ca^12^ and Mg–6.4Zn–0.2Ca–0.2Mn^9^. There are some RE-containing Mg alloys that exhibit higher yield strengths, like the Mg–8.2Gd–3.8Y–1Zn–0.4Zr, for instance^13^, although again, BioMg250 possess higher ductility.

As shown in the Table 2, in a few prior studies, higher strengths than reported here can be achieved with a dilute Mg alloy with application of additional severe plastic deformation steps, such as extrusion, hot extrusion, and equal channel angular extrusion, a severe plastic deformation technique^56^. For instance, for Mg–0.1Ca and Mg–1Ca, which are also dilute, Al-free, RE-free alloys, yield strengths of 290 (with 13% ductility) and 377 (with 2% ductility) MPa, respectively were achieved^56^. Here, the strengths of BioMg250 are reported without any severe post-extrusion (Ext) steps. The room temperature ductility (9 to 21%), however, would be sufficient for any of these severe forming steps and even higher strengths to be possible^35^.

We have established that the strength of this dilute Mg alloy is outstanding, a property that we attribute to the formation of very fine nanoscale precipitates during material processing. Toward understanding their contribution, the material was annealed at 400 ºC for 1 h, in order to remove the finest nanoscale precipitates. The suite of tension and compression mechanical tests was repeated on the AN material and the results are shown in Fig. 3. In all test directions and in tension and compression, the AN material had lower yield stress and peak stress, and higher strain to failure than the PA material for the same test (see Tables 2 and 3). After annealing, the plastic anisotropy seen in the PA alloy, however, persisted. In the AN material, the order in strength from highest to lowest is RD > DD > TD in tension, and ND > RD > TD in compression, as in the PA material. This similarity can be attributed to the fact that their starting textures and misorientation distributions were similar.

Notably with the annealing treatment, the ductility increased to 32% for all directions, and the plastic anisotropy and T–C asymmetry reduced to 0.86 yield and 0.64 peak for RD and these values are 0.67 and 0.71 for TD, respectively (see Fig. 3b,d,f). All three changes are signs of enhanced toughness and homogeneous deformation. These attributes make it suitable for the purposes of subsequent metal forming. In a prior study, the same annealed BioMg250 was found sufficiently formable for lamination with Nb via an accumulated roll bonding (ARB), a severe plastic deformation process^63^. After > 300%, sheets of equal 50% volume fractions of BioMg250/Nb with individual 150 µm layers were manufactured^63^.

**Figure 5:**
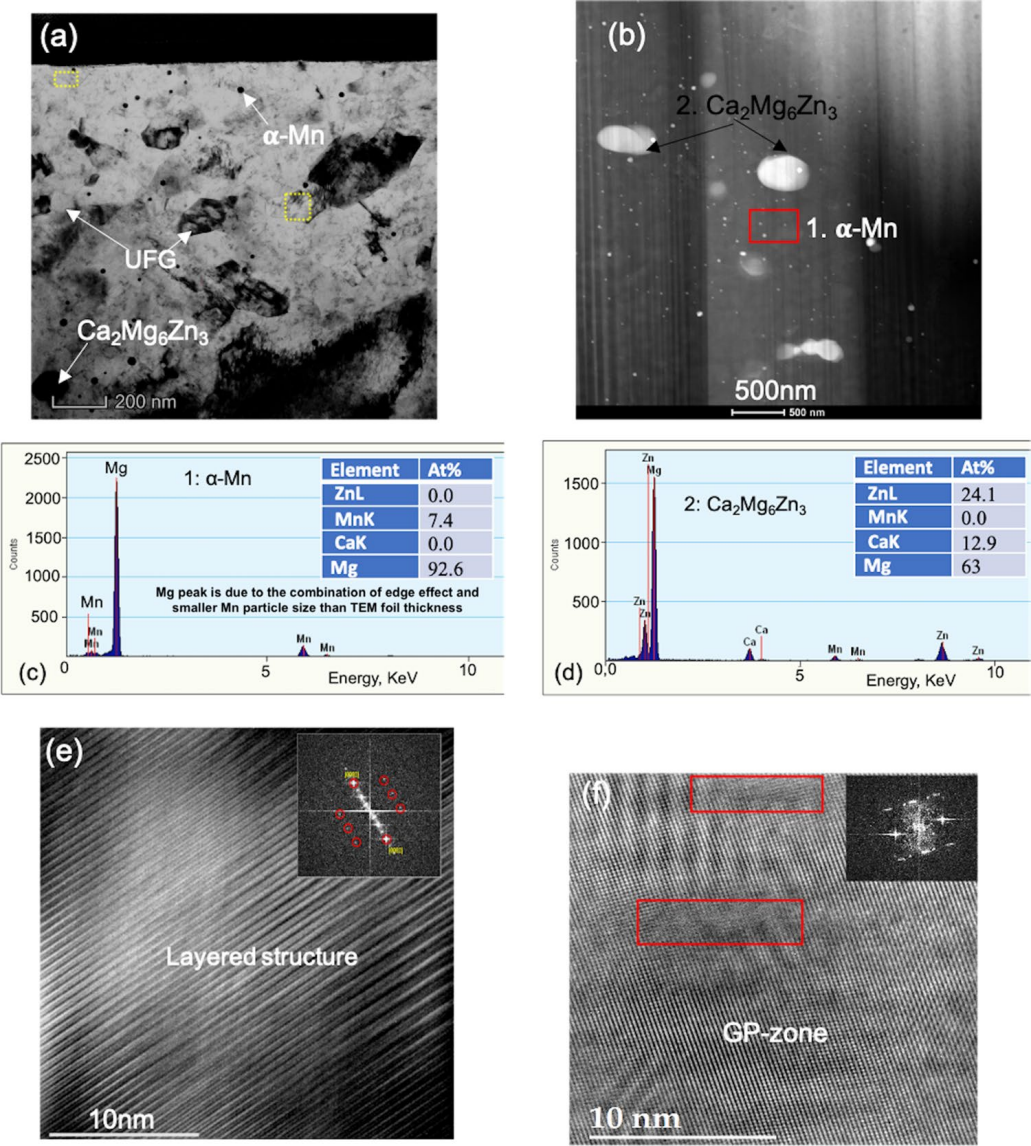
TEM images in the PA material showing the (**a**) co-existence of alpha-Mn (small), Ca_2_Mg_6_Zn_3_ (large) precipitates along with the 6H layered structure and submicron-size grains and (**b**) various precipitates. TEM-EDS scan showing (**c**) alpha-Mn (p#1), and (**d**) Ca_2_Mg_6_Zn_3_ (p#2), the (**e**) 6H layered structure, corresponding to the extra spots in the FFT patterns; (**f**) GP-zones (marked by the extra spot in the inserted FFT image) for the peak-aged specimens.

**Figure 6:**
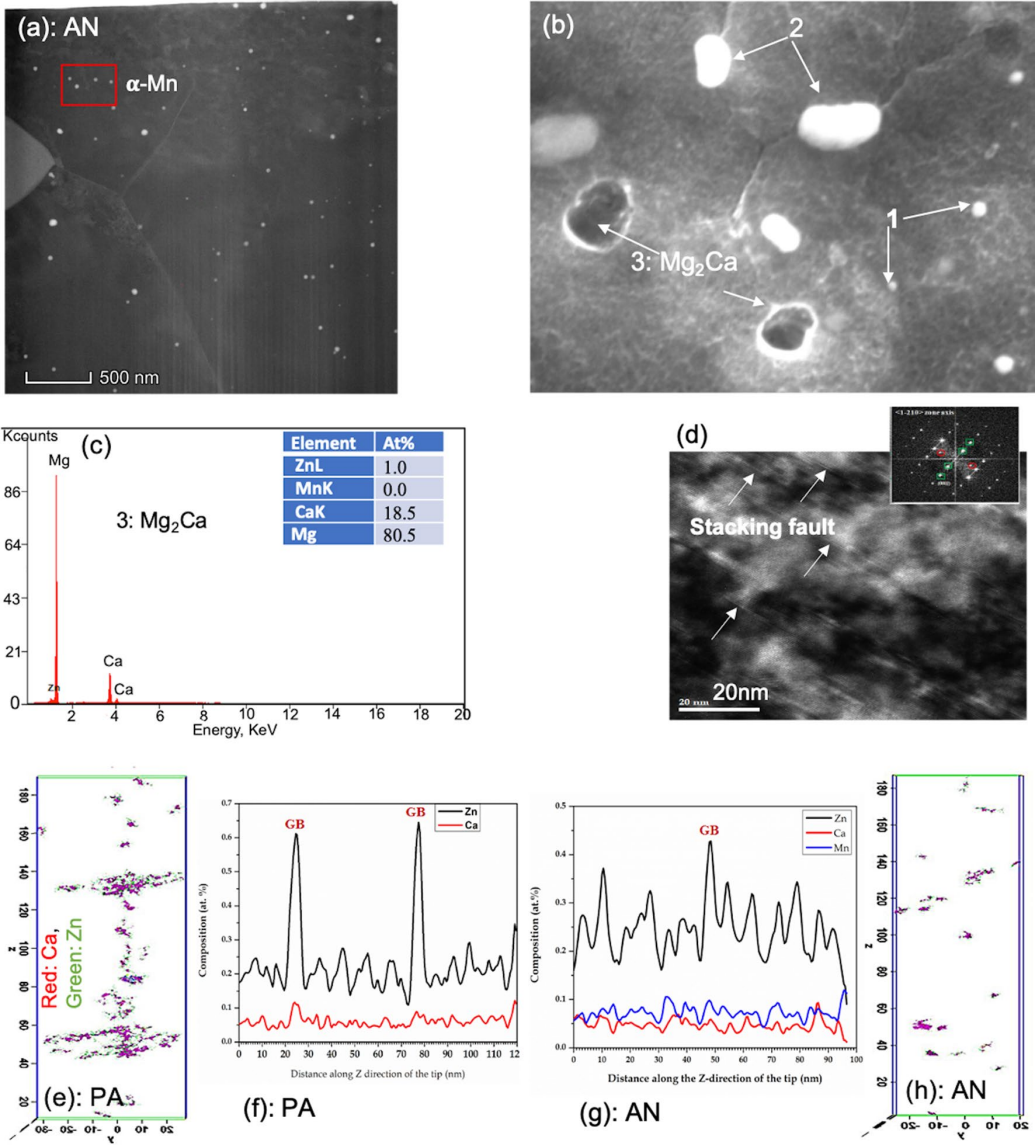
(**a**) TEM image showing the alpha-Mn precipitates in the annealed material, (**b**) SEM image showing the Mg_2_Ca (point 3) precipitates, confirmed by the (**c**) EDS scan, along with other two precipitates; (**d**) shows stacking faults (arrows) in the annealed material; (**e**–**h**) show the APT reconstruction of the Mn-Zn-Ca nanoclusters for the (**e**–**f**) peak-aged material, and (**g**, **h**) their dissolved state for the annealed material.

As a suspected source of strengthening in this dilute alloy, the nanoscale precipitates in the PA material were analyzed via TEM, HR-TEM, and ATP (see Figs. 5 and 6). First, we find that within the grains lay nearly uniformly distributed, nanometer-sized precipitates (see Fig. 5a,b). EDS scans of the TEM samples confirm that most of these precipitates are α-Mn precipitates (see Fig. 5c). The relatively weak peak intensity of α-Mn, or higher percentage of Mg content, may be due to the combination of edge-effects and a smaller Mn particle size (≈18 nm) than the TEM foil thickness (≈100 nm), resulting in signals both from the particles and the matrix. These α-Mn precipitates have been reported in many other Mg–Zn–Mn based alloys, although differing in alloying content than those identified in the alloy studied here^33,64^. Additional TEM analysis shows that many 10–50 nm sized α-Mn precipitates are clearly visible in both grain interiors and boundaries. Using multiple TEM scans, we find that overall the nanosizes of these α-Mn precipitates averaged 18 ± 12 nm, but ranging widely from 3 to 62 nm (see Table 4 and Fig. 5b).

**Table 4:** Precipitate morphology for the peak-aged and annealed material.

Condition	α-Mn, nm	Ca_2_Mg_6_Zn_3_, nm	Mg_2_Ca, nm	Zn-Ca solute clusters, nm
Peak-aged	18 ± 12	345 ± 170	285 ± 165	2–6
Annealed	52 ± 21	280 ± 89	1,095 ± 906	Dissolved

EDS scans of both the SEM and TEM images reveal relatively smaller, white submicron particles, (Figs. 5d, 6b) and relatively larger, dark submicron Mg_2_Ca particles (Fig. 6b-c). The former are possibly Ca_2_Mg_6_Zn_3_, based on phase diagram and other reports^3,11,33,60,64,65^. Unlike the α-Mn precipitates, these submicron particles, especially the Mg_2_Ca particles, lay primarily in the grain boundaries, with very few located within the grain interiors (see Fig. 1). Further TEM imaging, coupled with EDS analysis, reveals 100–300 nm sized Ca_2_Mg_6_Zn_3_ precipitates decorating the grain boundary and triple junctions (see Table 4 and Figs. 1,5,6).

Even higher resolution microscopy reveals the presence of a number of unique nanoscale structures. HR-TEM, combined with spot analysis between the corresponding (0002) and (0000) FFT patterns, shows that the material contains 6H multi-layered structures (Fig. 5e and SFig. 2) on the basal planes. These nano-sized precipitates are known for bearing a long-period stacking order, where the H stands for their characteristic hexagonal Bravais lattice^66^. Normally, for solutes with larger radii, like REs, and Sr, higher-order 10-14H structures are observed, but for smaller solute atoms, like Al, Zn, Cu, lower order structures are found^66^. Here, the APT analysis indicates that composition of the low order 6H structures is a metastable Mg_7_(Zn,Ca)_3_, unlike those reported in other studies. At this high resolution, coherent GP-zones were also observed in the basal plane (Fig. 5f). Using APT, we find a fine distribution of nanometer-size Zn-Ca solute clusters (2–5 nm) lay in the grain interiors (Fig. 6e-f), a finding that would not have been captured by HR-TEM, along with segregation of Zn and Ca to the grain boundary (see APT composition profile, Fig. 6f). APT results clearly identify segregation of both Zn and Ca in the PA alloy, whereas segregation occurs to a lesser extent in the AN alloy due to enhance diffusion at 400ºC annealing (see Fig. 6e–h). Solute segregation at the grain boundary not only prevents the preferential growth of the grain boundaries, it also exerts a drag effect on the grain boundary mobility and pin the boundary. Pinning effect of grain boundary is further modified by the presence of nm scale precipitates in the grain boundary. Therefore, although a comparatively higher annealing temperature and time is used for the AN material here, the extent of grain growth is not that high compared to previous reports of recrystallization and grain growth of other Mg–Ca–Zn alloys^46,67^.

Employing the same highly resolved microscopic analysis on the AN material shows that the precipitate and particle structures were substantially altered in structure, size, distribution, and chemistry. The α-Mn precipitates were again found within the grains of the AN material, but at much lower frequency and nearly triple in size (≈ 52 ± 22 nm) and ranging from 10 to 105 nm (see Table 4 and Fig. 6a). The annealed condition also contained submicron Ca_2_Mg_6_Zn_3_ and Mg_2_Ca particles, which primarily lay in the grain boundaries. SEM analysis shows that compared to the PA material, the Ca_2_Mg_6_Zn_3_ particles are smaller and fewer, while the size and number density of the Mg_2_Ca particles are larger (see Table 4 and Fig. 1).

The most substantial differences between the microstructures of the PA and AN materials were found at the finest scale. HR-TEM analysis finds that the annealed material contains many grains filled with basal stacking faults (Fig. 6d). Unlike the peak-aged material, no basal 6H layered structures and no GP-zones were observed (see SFigs. 2 and 3) that dissolves the metastable Mg_7_(Zn, Ca)_3_, layered structures and starts to produce stacking faults. As another significant difference, APT indicates nearly complete dissolution of the Zn in the annealed specimens, with very few Mn–Zn–Ca solute clusters (Fig. 6g, h).

## Discussion

In this study, the plastic anisotropy and tension–compression asymmetry in the yield stress, peak strength, and percent strain to failure in an Al-free, RE-free, dilute Mg alloy was studied using mechanical testing combined with a suite of characterization techniques EBSD, SEM, TEM, HR-TEM, and APT. The alloy exhibits remarkable strength, despite its low concentration of alloying elements (≈ 2 wt.%) and lack of rare-earth elements. Its achievement of simultaneous high strength and reasonable ductility can be attributed to its hierarchical microstructure, starting with a bimodal distribution of coarse grains and ultrafine grains and submicron grain boundary particles, and down to the nanoscale precipitates and 6H structures, and finally to the fine < 5 nm-sized GP zones and solute clusters distributed throughout the grains.

For dilute Mg alloys of this type, the anticipated sources of strengthening are grain refinement and the formation of very fine nanometer-scale Zn-containing precipitates. Removing these two microstructural features via annealing yields a material that is half as strong. The granular structure had an ultrafine grain structure, with a majority of the grains being submicron in size, which is well known to lead to stronger materials^59,68^. Further, the grain structure was highly inhomogeneous, containing 47.5% grains with diameters less than 5 µm, 47.5% less than 1 µm, and only 5% that were large micron-sized grains. Many reports recently have suggested that hierarchical structures can give rise to stronger materials^56,68^. The hierarchical structure here was substantially altered after annealing (at 400ºC/1 h), becoming homogeneous and comprised of nearly equiaxed grains of sizes of approximately 13 µm (see Table 1 and Fig. 1), and undoubtedly contributing to the observed 50% reduction in strength.

While we cannot dismiss the ultra-fine grain sizes, dominating the material microstructure, as contributing to the high strength of the PA alloy, it is also important to acknowledge the possible role that the multitude of nanostructured Zn-containing precipitates play in strength and plastic anisotropy. In this high-strength dilute alloy, many special nanostructured precipitates, apart from G-P zones, were found, such as the 6H basal layered structures and 2–5 nm Zn–Ca solute clusters (see Figs. 5e and 6e,f). In the annealed (weaker) material, none of these structures were found. While a variety of precipitates were observed, they were bigger in size and lower in density than these precipitates, and without Zn. GP zones and the 6H precipitates lie on the basal planes, and thus, would act as obstacles to dislocations gliding on intersecting prismatic and pyramidal planes. The fine Zn–Ca solute clusters and the ellipsoidal-shaped 5 nm and the larger 40 nm *α*-Mn precipitates are uniformly dispersed in the grains, and would resist glide by all slip families in the strained crystals. Since these precipitates contain Zn, the matrix Mg is relatively free of Zn, which could increase lattice resistant to dislocation glide compared to an Mg matrix containing Zn. Atomistic simulations have shown that addition of Zn in Mg lowers the Peierls barrier and broaden the equilibrium core size of basal dislocations^69^.

The combined HRTEM and APT analysis suggest how the 6H-layer structures on the basal plane of the Mg may have formed. Prior DFT studies have implied that the 6H-type layer structure could form via the segregation of Zn and Ca, within the range of 8–25 at.%, to the I_1_-type SF^66^. Here, the HRTEM analysis provides physical evidence of fine nm scale 6H-layer structures, and the APT data indicate that their composition is Ca_2_Mg_6_Zn_3_, distinct from that of other, even thicker layered structures in other higher concentration alloys and alloys with larger radius solutes^66^. Further, the same analysis finds no H-layered structures but only I_1_-type SFs in the annealed sample (see Fig. 6d, and SFig. 3). As clearly seen in HRTEM images and its corresponding FFT patterns (see SFigs. 2 and 3), during annealing at 400℃ for 1 h, Zn and Ca have diffused out from the 6H-layer structure, leaving behind I_1_-type SFs. Taken together, this work implies that the 6H structures were stabilized through Zn and Ca segregation to the I_1_-type SFs, which occurred during the thermo-mechanical steps involved in manufacturing this dilute alloy.

While annealing lowered the tensile yield strength of the alloy by 50%, compared to the PA, it increased substantially the room temperature ductility and lowered both the T-C asymmetry and plastic anisotropy. The ductility of Mg alloys is normally improved in a number of ways, either by lowering the texture, possibly reducing the propensity for twinning, or the addition of alloying elements that help in activating < c + a > dislocations to promote plastic deformation along the c-direction of the Mg crystals^1, 2^.

Generally, pure Mg and its alloys (like AZ31) exhibit (0001) strong basal texture after deformation processing. Although both the peak-aged and annealed samples here possess this expected (0001) ND basal texture, the maximum texture intensity is 3.8 m.r.d., which is substantially weaker, and the basal poles are slightly tilted towards the TD. This low texture intensity of both materials seen in Fig. 2 compared to other Mg alloys like AZ31^40–42^, is one of the primary reasons for the higher ductility of this dilute alloy. Yet, because they exhibit similar textures, texture weakening is not reasoning the annealed material possesses a significantly enhanced ductility.

The analysis here combined with prior atomistic calculations suggest that < c + a > dislocation is more active in the annealed material, which would also explain its higher ductility. Evidence exposed by HRTEM analysis of the annealed material implies that the I_1_-type SF may have helped in nucleating mobile < c + a > dislocations (see SFig. 3. Further, APT data also suggests that the amount of Zn solute in the matrix increases after annealing whereas grain boundary segregation decreases. As mentioned, atomistic calculations indicate that the resistance to basal slip is reduced with Zn additions. Further, similar type of calculations reported that addition of Zn softens the resistance to pyramidal slip^70^. SEM fractographs of the tensile tested specimen further strengthen this argument, finding a predominance of basal slip traces in the peak-aged alloy (see SFig. 4a), while a predominance of pyramidal slip traces in the annealed samples (see SFig. 4b).

In summary, we study the room temperature structural properties of BioMg250, a rare-earth- and aluminum-free Mg alloy, containing trace amounts of Zn, Ca, and Mn (only about 2% by wt.). Room temperature tensile tests show a remarkably high 0.2% yield strength in the range of 214–267 MPa, and ultimate strengths of 307 MPa, while maintaining impressive ductility in the range of 9–21%. The compressive yield strength varies from 166 to 271 MPa. The combination of strength and ductility exceeds most Mg alloys today, despite its dilute alloying content. We show that the material has a highly heterogeneous microstructure, comprised of a bimodal grain structure containing ultra-fine, partially recrystallized grains, along with a highly density of nanometer-sized, alpha-Mn precipitates, 2–5 nm Zn–Ca solute clusters, and coherent GP-zones within the grain interiors. Annealing the material to remove these features leads to substantially reduced the yield and ultimate tensile strengths by 55% and 22%, respectively. It is, however, notable that, despite this reduction, the strength of the annealed material is still comparable to that of many structural, dilute Mg alloys, while exhibiting much greater ductility of 32% in all three directions tested. The study here demonstrates that BioMg250 has the light, RE-free, Al-free composition and high strength to serve as a biocompatible, biodegradable structural material candidate for both biomedical and automobile industries.

## Methods

The BioMg250 was synthesized by melting elemental Mg (balance) with 1.2 Zn, 0.4Ca, and 0.4 Mn (by wt.%). The solidified material was then hot-rolled to a total rolling reduction of 53% for a final plate thickness of ≈ 2 mm. The plate is then peak-aged at 200ºC for 2 h, which we refer to as the peak-aged condition. More details of the peak-aged processing conditions are not revealed for proprietary reasons. In this work, the as-received peak-aged plate was further annealed at 400ºC for 1 h in a box furnace under atmospheric condition followed by water quenched, which we refer to as annealed condition.

Specimens from both peak-aged and annealed materials were manufactured by electrical discharge machining (EDM) for use in various microstructural and mechanical characterizations. Both the electron backscatter diffraction (EBSD) and SEM samples underwent subsequent standard metallographic preparation before characterization (see details in supplemental section). EBSD patterns were collected using a Pegasus system (Octane Plus SDD detector and Hikari High Speed Camera) in a Tescan Lyra 3 GMU FE-SEM with an accelerating voltage of 25 kV and step size of 0.50 μm. This was done to measure grain morphologies, crystallographic texture, and grain boundary and twin boundary misorientation angles. SEM was also used to observe grain morphology, inclusions and produce fractographs of the tensile/compression fractured surfaces, while energy dispersive spectroscopy (EDS) was used to identify the elemental compositions of the precipitates. For the SEM grain observations, the major (l) (generally along rolling directions) axis was reported as grain size. APT, TEM and HR-TEM were used to observe the nanostructures, like precipitates, stacking faults/layered structures, GP zones from the peak-aged and annealed specimens. Focused ion-beam (FIB) was used to lift-out and prepare TEM foils and APT needles.

Uniaxial tensile testing at room temperature was performed on the EDM-cut, flat dog-bone shaped sub-sized SSJ-2 (16 × 4 × 0.5 mm with a gauge section of 5.0 × 1.2 × 0.5 mm, see SFig. 1. As shown in SFig. 1, the tensile axis was aligned in one of three different loading directions with respect to the rolled plate: rolling direction (RD); 45º or diagonal direction (DD); and transverse direction (TD). Tensile tests were carried out on an MTS 810 servo-hydraulic universal testing machine and at a displacement rate of 0.30 mm/min, equivalent to a strain rate of about 10^−3^/s. At least 3 tests were conducted for each direction for both alloy conditions. The tensile properties were determined in accordance with ASTM test standard E8M-15a. Room temperature compression testing was also conducted at three different orientations: rolling direction (RD), normal direction (ND), and transverse direction (TD). The dimensions of the compressive cylindrical specimens are restricted by the plate thickness, which is approximately 2 mm. While the RD and TD specimens nominal dimensions are 2 mm in diameter and 4 mm in height, the ND specimen dimension is 2 mm × 2 mm (SF. 1). All compression tests were performed on the same MTS 810 machine, with a strain rate equivalent to 10^−3^/s. At least, three samples were deformed per category to ensure repeatability and accuracy of the measured data. Compression test loads and displacements were measured in accordance with the ASTM E-09-19 standard.

## Supplementary information

Supplementary file1
