# Supplementary material for: Rare-earth- and aluminum-free, high strength dilute magnesium alloy for Biomedical Applications
Source: Sci Rep. 2020 Sep 28;10:15839. doi: 10.1038/s41598-020-72374-z (PMC7522977; doi:10.1038/s41598-020-72374-z)
Supplement: Supplementary file 1 — Supplementary file1 [file 41598_2020_72374_MOESM1_ESM.pdf]

Rare-Earth- and Aluminum-Free, High Strength Dilute Magnesium Alloy

Md Ershadul Alam, Soupitak Pal, Ray Decker, Nicholas C. Ferreri, Marko Knezevic, Irene. J. Beyerlein

Supplemental Figures (SFigs.)

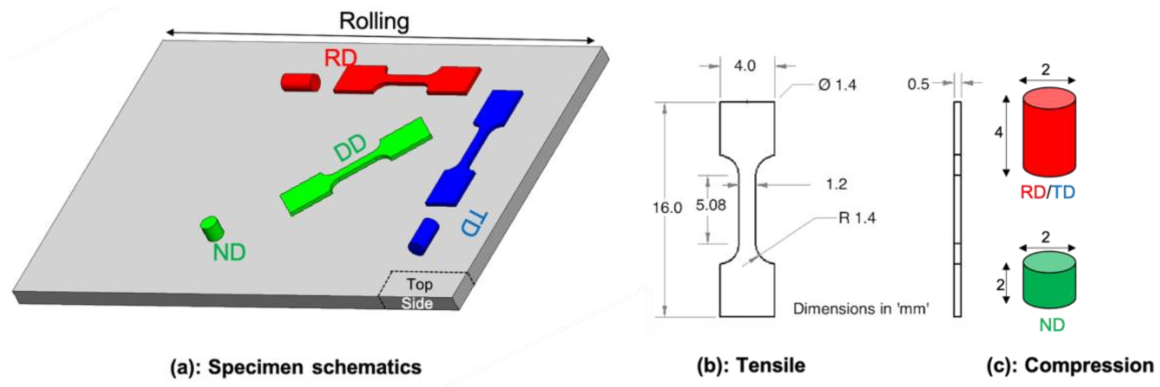

SFig. 1 Schematics of (a) plate and specimens test orientation with respect to the rolling direction; (b) tensile; and, (c) compression specimens.

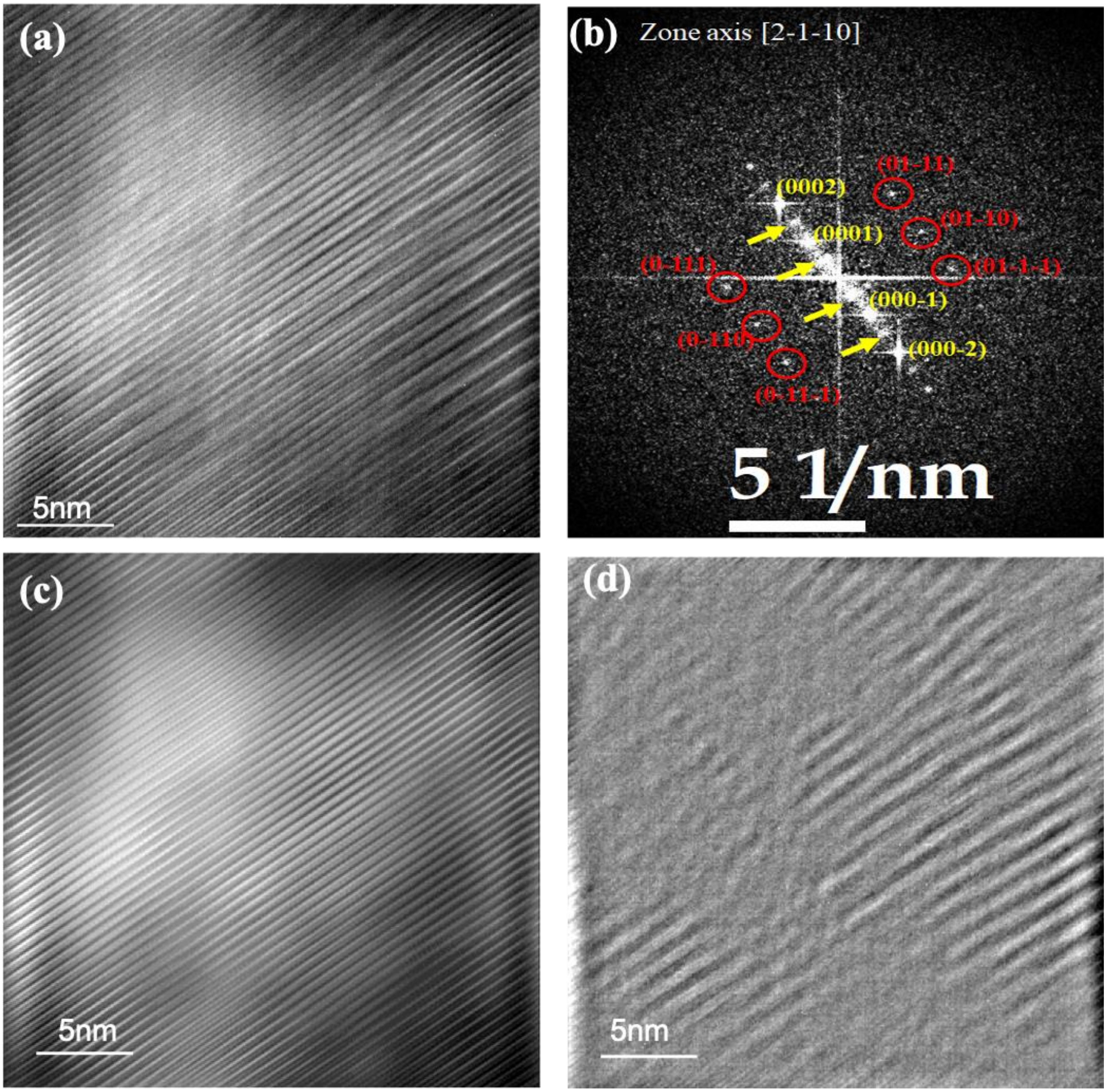

SFig. 2 Fig. (a) showing the HRTEM image of the layered structure for PA specimen; (b) FFT pattern showing the foil normal is [2-1-10]. Extra spots originated in between (0002) and (0000) are indicated by yellow arrows; Figure (c) and (d) showing the inverse FFT image of the image 'a' after removing the extra spots (c), and with the extra spots (d), respectively. Fig. (d) clearly indicates extra spots in the FFT pattern of Fig. (b), associated with the layer structure formation of 6H type.

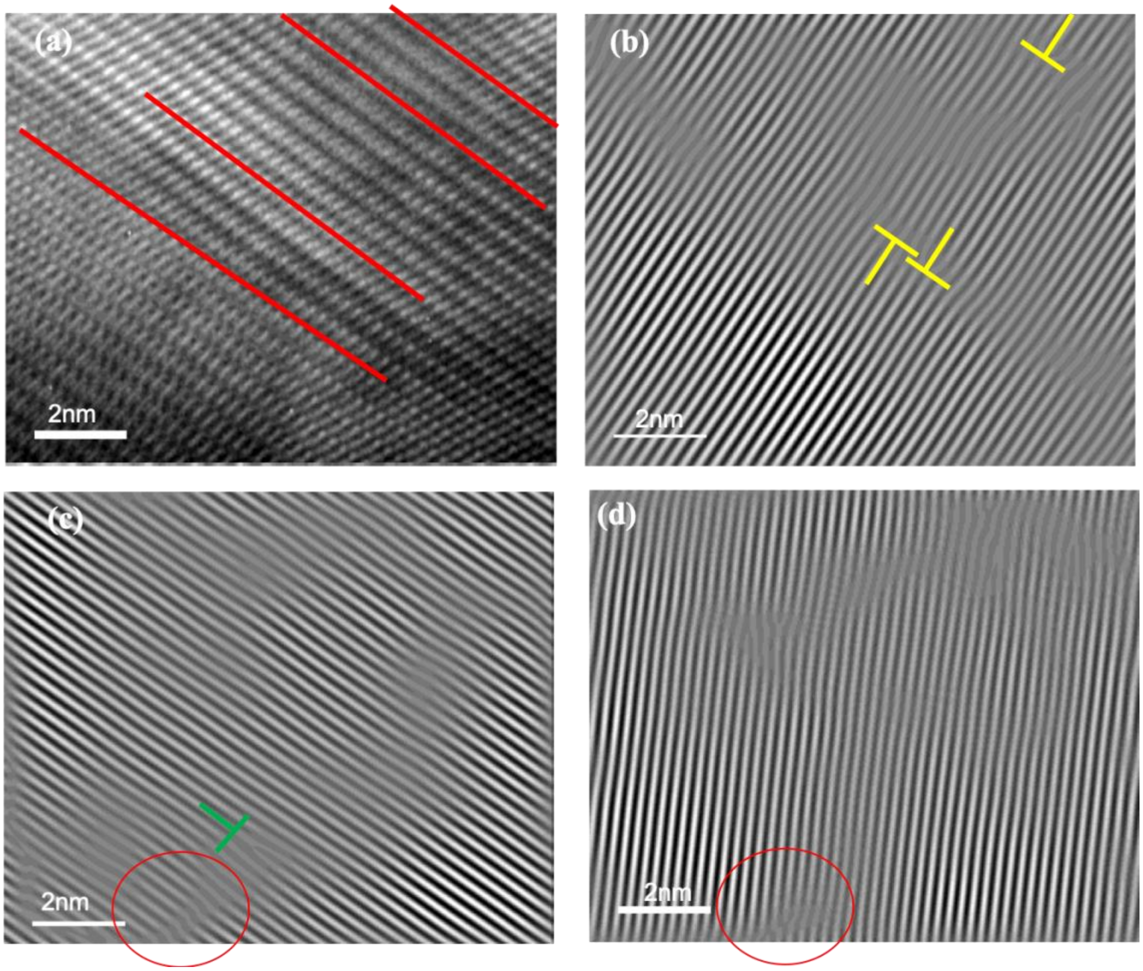

SFig. 3 Fig. (a) showing the HRTEM image of annealed BioMg250. Faulted regions are marked with red lines. By applying the g.b ≠ 0 and g.bxu ≠ 0 for visibility of dislocations: (b) image formed using the spots (01-10), the 1/3<1-100> type dislocation are shown; (c) image formed using (0002) spots, the <c>-type dislocation are marked by green and <c+a>-type dislocations are marked by red circle; and (d) using (0-111) spots, the red circle shows <c+a> type dislocation.

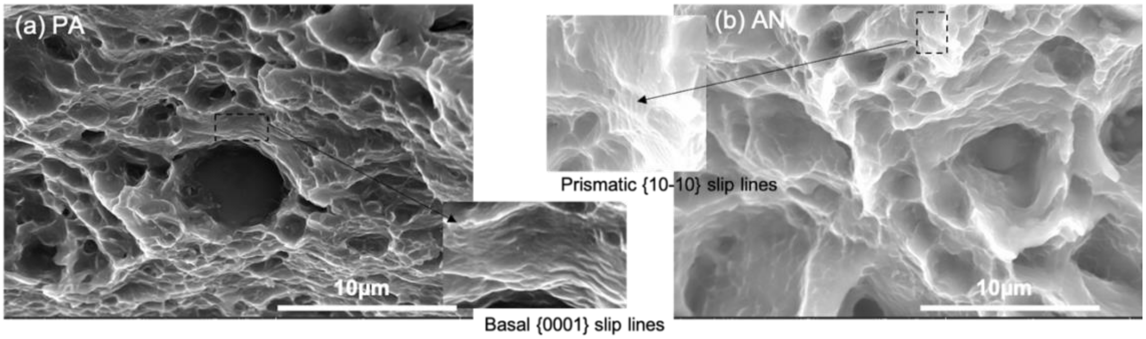

SFig. 4 SEM tensile fractographs showing (a) smaller dimples size with basal-type slip lines for peak-aged samples (flatter, see insert); and (b) larger dimples with wavy-like prismatic slip lines for annealed samples (see insert).

Supplemental Notes

Methods

Materials Preparation: The BioMg250 was synthesized by melting elemental Mg (balance) with 1.2 Zn, 0.4Ca, and 0.4 Mn (by wt.%). The solidified material was then hot-rolled to a total rolling reduction of 53% for a plate thickness of ≈ 2mm. The plate is then peak-aged at 200ºC for two hours, which we refer to as the peak-aged (PA) condition. More details of the peak-aged processing conditions are not revealed for proprietary reasons. In this work, the as-received peak-aged plate was further annealed at 400ºC for 1h in a box furnace under atmospheric condition followed by water quenched, which we refer to as annealed (AN) condition.

Microstructural Characterization: Specimens from both peak-aged and annealed materials were manufactured by electrical discharge machining (EDM) for use in various microstructural and mechanical characterizations. Both the electron backscatter diffraction (EBSD) and scanning electron microscopy (SEM) samples underwent subsequent standard metallographic preparation before characterization. These specimens were mechanically ground using a sequence of 180, 240, 400, 600, 800, and 1200 grit SiC abrasive paper (Allied High Tech Products) followed by a sequence of 9, 3, and 1µm Al_2O_3 abrasive paper (Precision Surfaces International). After grinding, samples were further polished using an aqueous (deionized) 0.3 µm Al_2O_3 suspension followed by an aqueous (deionized) 0.05 µm Al_2O_3 suspension both on non-abrasive soft cloths. Due to the soft nature of Mg, a final 5-second etch was needed to further improve sample surface quality and EBSD pattern clarity. The etchant used was a solution containing 2% (by volume) concentrated nitric acid in deionized water.

EBSD patterns were collected using a Pegasus system (Octane Plus SDD detector and Hikari High Speed Camera) in a Tescan Lyra 3 GMU FE-SEM with an accelerating voltage of 25 kV and step size of 0.50 μm. This was done to measure grain morphologies, crystallographic texture, and grain boundary and twin boundary misorientation angles. EBSD data was analyzed using both TSL OIM software and the MTEX toolbox in MATLAB.

Scanning electron microscopy (SEM) was also used to observe grain morphology, inclusions and produce fractographs of the tensile/compression fractured surfaces, while energy dispersive spectroscopy (EDS) was used to identify the elemental compositions of the precipitates. For the SEM grain observations, the longest (l) and shortest (s) dimensions of minimum 500 individual grains were tabulated using 'ImageJ64' software, and the longest (generally along rolling directions) dimensions were reported. The grain aspect ratio (GAR) was measured by l/s for all grains. Atom probe tomography (APT) and transmission electron microscopy (TEM) were used to observe the nanostructures, like precipitates, stacking faults/layered structures, GP zones from the peak-aged and annealed specimens. Focused ion-beam (FIB) was used to lift-out and prepare TEM foils and APT needles.

Mechanical testing: Uniaxial tensile testing at room temperature was performed on flat dog-bone shaped sub-sized SSJ-2 specimens. These samples were fabricated via electrical discharge machining (EDM) with total dimensions of 16 x 4 x 0.5 mm in length, width, and thickness, respectively, and containing a gauge section of 5.0 x 1.2 x 0.5 mm [1]. As shown in Supplemental Figure SF. 1, the tensile axis was aligned in one of three different loading directions with respect to the rolled plate: rolling direction (RD); 45º or diagonal direction (DD); and transverse direction (TD). All tensile specimens were then sanded with 1500 grit to remove surface oxides and contamination, minor cracks and local residual stresses due to the EDM process. Tensile

tests were carried out on an MTS 810 servo-hydraulic universal testing machine and at a displacement rate of 0.30 mm/min, equivalent to a strain rate ≈ 10^{-3}/s. At least 4 tests were conducted for each direction for both alloy conditions. The tensile properties were determined in accordance with ASTM test standard E8M-15a [2].

Room temperature compression testing was also conducted at three different orientations: rolling direction (RD), normal direction (ND), and transverse direction (TD). The dimensions of the compressive cylindrical specimens are restricted by the plate thickness, which is ≈ 2 mm. While the RD and TD specimens nominal dimensions are 2 mm in diameter and 4 mm in height, the ND specimen dimension is 2 mm x 2 mm (Fig. 1c). All compression tests were performed on the same MTS 810 machine, with a strain rate equivalent to 10^{-3}/s. However, the machine was equipped with a customized compression fixture consisting of two cylindrical compression dies aligned vertically along the axis of the actuator. The compression dies are made of Inconel with smooth, flat surfaces. Tungsten carbide (WC) bearing blocks (5 mm thick, 20 mm diameter), also flat-surfaced, were used at both sides of the dies to protect the face of the Inconel dies and to help transmitting axial load. Boron nitride spray-II was used to minimize the effects of contact friction between the faces of the specimen and the upper and lower compression dies and blocks. All the dies and the blocks were perfectly aligned before the tests and were found parallel to the loading axis after testing. At least, four samples were deformed per category to ensure reputability and accuracy of the measured data. Compression test loads and displacements were measured in accordance with the ASTM E-09-19 standard [3].

References:

1. Alam, M. E., Pal, S., Fields, K., Maloy, S. A., Hoelzer, D. T. & Odette, G. R. Tensile deformation and fracture properties of a 14YWT nanostructured ferritic alloy. *Mater. Sci. Eng. A* **675**, 437–448 (2016).
2. ASTM E8M-15a. ASTM E8/E8M - 15a: Standard test methods for tension testing of metallic materials. in *Annual Book of ASTM Standards* (ASTM International, 100 Barr

Harbor Drive, PO Box C700, West Conshohocken, PA 19428-2959, United States, 2015).
3. ASTM-E9-19. Standard test methods of compression testing of metallic materials at room temperature. in *Annual Book of ASTM Standards* (ASTM International, West Conshohocken, PA, 2019).
